# Pathology of the Nervous System

**Published:** 1836-10-01

**Authors:** 


### Medico-Chirurgical Review,

### No. L.

[No. 10 of a Decennial Series.]

JULY 1, TO OCTOBER 1, 1836.

### PATHOLOGY OF THE NERVOUS SYSTEM.

I. LECTURES ON THE NERVOUS SYSTEM AND ITS DISEASES. By *Marshall Hall*, M.D. F.R.S. &c. Octavo, pp. 171. London, 1836.

II. OUTLINES OF HUMAN PATHOLOGY. By *Herbert Mayo*, F.R.S. &c. Art. Of the Nerves.

THE physiology of the nervous system has deservedly attracted the attention of the profession in a high degree. It involves more than that of any other system in the body, the consideration of the higher vital laws, and, while it partakes of their obscurity, it participates also in their philosophical interest.

It is not many years since, direct experiment being applied to the solution of the functions of the nerves, a sudden burst of discovery ensued, as brilliant as it was unexpected. It is not surprising, if the truths that were elicited led men to suppose, that the extension of experiments would decide the functions of all parts of the nervous system, as well as of a few. The natural result ensued. Experiments were wantonly and absurdly resorted to, and, after the most complicated butchery, the physiologist pretended to deduce some simple and certain consequences. The fallacy became, on reflection, apparent, and such experiments soon ceased to attract any notice but disgust.

We do not know that direct experiment has yet done all that it is able to do, for the elucidation of the functions of the nervous system. It would be presumptuous to say that. But it has done all that it seems capable of doing at present, and we may judiciously pause and enquire if other means of analysis are in our reach.

Careful and deliberate observation of phenomena, the investigation of the comparative anatomy of the nerves, and the study of their pathology, all promise to assist us materially. What has been actually discovered has not been accurately arranged. The feverish excitement generated by the rapid results of experiment, has hardly subsided into the calm and comparatively slow process of generalization and induction. Now is the time for comparing the results of all investigations—of symptoms—and of pathology, as well as of experiment.

Experience has shewn that the clever experimentalist is not necessarily the close reasoner. Experiment, indeed, begets exactness, but exactness is sometimes the source of all fallacy. Demonstrative evidence is, of course, the best, and should be demanded where the case admits of it. But when the case does not admit of it, there is no error so monstrous or so dangerous as the obstinate attempt to apply the rules of demonstration to it. Such, however, has been done with respect to the nerves. As division of the portio dura shews its office, it has been supposed that division of the sympathetic should shew *its* office. It has not done so, and it has almost been insinuated that the sympathetic has no office at all. In an elementary work on physiology, the functions of the ganglionic nerves are not even hinted at, or if hinted at, that is all. The excuse of the author is of the kind we have described. He is an experimentalist—must have direct evidence and demonstration—in the instance of the ganglionic nerves he has neither—so he rejects a whole mass of reasoning, and of accumulative evidence, and morally burkes the system altogether. Such are the inconsistences of persons who affect extreme exactness. This very author believes in imbibition, and thinks that the tissues were made like a leaky boot—not waterproof. Reasoning is against this; but then there was in its favour an experiment, an apocryphal experiment. Reasoning, of course, kicked the beam.

We shall not, on this occasion, enter on the physiology of the nervous system. We shall advert to that in a succeeding number. At present we shall give a sketch of its pathology. That sketch will be taken from the two works whose titles head this article. Both are the productions of men of ability, but of ability of very different descriptions. Mr. Mayo is a bold reasoner—Dr. Hall is a subtle one—the former generalizes hastily—the latter is too much disposed to refine. Yet both, as we observed, are men of ability, and we shall proceed to examine their accounts of the nervous system.

Dr. Hall's work consists of twelve lectures. The first is dedicated to the anatomy and physiology of the nervous system, which, for the present, we eschew—the remainder treat in succession of congenital states of the nervous system, and of its diseases in early life—of the diseases of the nervous system in adult age—of cerebral diseases resulting from various affections of the system—of the diseases of the spinal marrow—and of the diseases of the spinal motor nerves.

The arrangement of Mr. Mayo is this. He considers seriatim, the diseases of the nerves as organs of consciousness—the diseases of them in reference to nutrition and secretion—the diseases of the spinal cord—the diseases of the encephalon.

The classification of each is founded more or less on physiological considerations. Those of Dr. Marshall Hall are peculiar. As we have already mentioned, we avoid on this occasion all allusion to them, and present only pathological facts, intermixed with what therapeutic consequences may be found to flow from them. It is our business to dovetail the two authors as cleverly as we are able, and to solder them with a little critical lead where they refuse a more intimate connexion.

We commence with the second lecture of Dr. Hall. It is on—

### The Pathology of the Nervous System.

In order that our readers may follow this gentleman, we must inform them in limine that he divided the nervous system into—

I. The cerebral, or the sentient and voluntary;
II. The true-spinal, or the excito-motory; and
III. The ganglionic, or the nutrient, secretory, &c.

The first and the third divisions will be readily understood, and are sufficiently familiar. But what is the excito-motory section of the nervous system? It is this. If a stimulus be applied to a part supplied and regulated by the spinal nerves, the impression is conveyed by sentient nerves *to* the spinal axis, and the action consequent upon it is re-conveyed *from* the spinal axis by motor nerves to the original part. All this is to a certain degree independent of the brain, and therefore Dr. Hall has adopted the term, and attaches much importance to the thing. We make this explanation at once, that our readers may perfectly comprehend our author when he speaks of the excito-motory system.

One other remark appears to be necessary. Both the works before us are to a certain degree elementary. We have no intention of dwelling on what is generally admitted, and therefore we shall not, cannot offer a systematic analysis of either work. We shall select particular points, and those only, for extract, analysis, or commentary, as we may think fit. The form of publication adopted by our authors imperatively requires this arrangement.

Our simplest idea of the pathology of the nervous system, consists in supposing any affection of it to be either an exaltation or a diminution of the functions performed by any of its parts. In the sentient nerves sensibility may be increased or lost; in the motor nerves there may be incessant action, as permanent contraction of the muscles—or irregular action, as spasm—or want of action, as paralysis; the functions of the brain may be excited into delirium, perverted in many ways, or sunk in coma; the cerebral axis may cease to apprehend impressions, as when it is crushed—or it may exaggerate them, as when inflamed—it may fail to convey its stimulus to the muscles—or it may send an undue amount, as in priapism.

Let us look at one or two of the facts connected with the pathology of the cerebrum.

### Palsy.

It has long been known, that disease in one hemisphere of the brain induces paralysis on the opposite side of the body. Dr. Hall remarks that it has been fully ascertained that disease confined to one hemisphere of the cerebrum, or of the cerebellum, and to one side of the mesial plane in the tuber annulare, constantly affects the *opposite* side,—whilst disease, confined to one of the lateral columns of the medulla oblongata and medulla spinalis, affects the *corresponding* side, of the muscular system. The encephalon has a *crossed-effect;* the medulla a *direct effect.*

Let us now turn to Mr. Mayo. The consideration of palsy follows that of apoplexy in his arrangement.

Palsy, he says, from a single lesion of the brain, affecting only one side of the body, should be termed hemiplegia. This is a question of terms; but we prefer sticking to the old. Palsy is the general term—hemiplegia the particular. If he discards palsy, what substitute will he find for it? A substitute he must find, for it would be awkward to talk of hemiplegia of the portio dura, of the optic nerve. Besides, particular nerves may be palsied, independently of affection of the cerebrum. We repeat that it is an immaterial question of terms, but, so far as our own tastes go, we see no necessity for cramping our vocabulary.

Hemiplegia, then, proceeds Mr. Mayo, is either general or partial; that is, it invades either the whole, or part only, of the side attacked. Hemiplegia is alternating, when it invades one side of the face, and the opposite side of the trunk and limbs.

Hemiplegia is either paralytic, anæsthetic, or complete; that is, it may extinguish either voluntary motion alone, or sensation alone, or both at once. Hemiplegia, again, is either perfect or imperfect; that is, it may either suppress or only weaken voluntary power and sensation.

To illustrate these positions, he relates in succession cases of paralysis of the right side of the face—of the right side of the face and right arm, and of general hemiplegia of the left side, which terminated fatally in coma. These cases need not detain us. But the one that follows is more interesting. It is an instance of anæsthetic hemiplegia of the left side of the body, with paralysis of the right side of the face.

*Case—July,* 1835. "Thomas Embury, ætat. forty-two, one morning after breakfast, now seven weeks ago, on stooping to open a plate-chest which had been made of green wood, and kept closed some time, was so powerfully affected by the close smell which issued from it (such is his account), as to fall backwards, but without losing his senses, or striking his head. On recovering, he found he had undergone a paralytic seizure: the left side of the face and head and body and limbs were numb; the left arm more so than the left leg: he believes that no muscular weakness of the left side was combined with the anæsthesia; but the leg he thinks, dragged a little during the next two or three days. In four or five days, however, the leg, whether paralysed at first or not, had recovered both strength and feeling. The arm has been slower in recovering feeling; and even now the little finger and ulnar edge of the hand and forearm are in a degree numb. The sensibility of the left side of the face is entirely returned. What may have been the state of the right side of the face on the day of the seizure he does not know; but the following morning he observed that it was paralyzed: he could not close the right eyelid; the mouth was drawn to the left side; the sensibility of the right side was unimpaired. The first day he had some difficulty in swallowing; and for three or four days his speech was thick and imperfect. After a few days he felt a pain, for an extent which a shilling would cover, deep-seated on the right temple, midway between the eye and upper part of the ear. This pain has continued, though now considerably mitigated, to the present time. He has occasionally confusion of thought. He experiences at times sensations like life-blood in the arm and legs, but no spasm. The right eye is weak, but not inflamed: when it is unclosed, he sees double, owing to a slight deviation of the right optic axis outwards." 198.

As hemiplegia may depend on various lesions, its proximate cause is obscure. Yet Mr. Mayo conceives that he has proceeded some little way in the solution of the *why*. Shall we record a triumph, or deplore a failure? Let us see.

"The solution of the question which I am disposed to adopt, and which I believe not to have been previously entertained, is the following:—I conjecture the immediate cause of hemiplegia to be a depressing influence or shock, originating in the brain when in certain states of lesion, and propagated from it to the medulla oblongata and spinal marrow.

It may be asked, whether it is reasonable to suppose that the brain, when in a state of lesion, *can* originate any such depressing or palsying shock? Reasoning upon analogy, it is probable that the brain *may* possess this property. It is certain that, upon other organs than those of animal life, the brain *does* exert such disastrous and depressing influence. For example, in persons of a nervous temperament, sudden and overwhelming intelligence will sometimes temporarily stop the heart's action. Physical impressions, again, such as crushing a part of the brain or spinal marrow, will temporarily palsy the heart; an experiment the more conclusive, that the heart's action will continue undisturbed during the entire removal of the brain and spinal marrow, if the abstraction of these parts be made with gentleness. Is it surprising that lesion of the brain, which can palsy the heart, the action of which is not derived from the nervous system, should throw palsy upon the voluntary muscles, that in so many ways are habitually influenced through it? Such a force of depression, similar in kind, although much less in degree, mental affections alone have the direct power of temporarily producing; the 'genua labant, tremor occupat artus,' the weakness of terror, is probably an instance of imperfect or temporary palsy so produced.

Upon these and other grounds, which will be presently mentioned, it appears reasonable to believe that a lesion of the brain is capable of originating a depressing force, which can strike with palsy the organs in the cord from which the nerves arise; or that palsy from cerebral disease is not caused by the interruption of an accustomed stimulus, but by the production of a new and withering influence, transmitted from thence to the origins of the nerves." 200.

It is obvious that the chief merit of any explanation of this sort is its clearness. The fact that it affects to solve is admitted—the quomodo is the point at issue. If obscurity attends the solution of that, it leaves us where it found us, or plunges us deeper in the dark.

Unless Mr. Mayo applies the term shock in its ordinary sense, it conveys of course no clear idea. We presume, then, that in such a sense it is employed, and that Mr. Mayo's theory supposes the brain capable of originating a sudden, violent, and injurious impression upon parts, which it may or may not habitually influence.

But the very term shock does not imply a continuous action, while the palsy occasioned by lesion of the brain is as constant as its cause. A small effusion of blood occurs in a cerebral hemisphere—hemiplegia instantly results. Gradually the blood is altered, absorbed—gradually the hemiplegia ceases. Does this look like a shock—"a withering influence"? Is it consistent with what we know of the economy, to suppose that an organ is gifted with the strange and monstrous function, of originating a new action only for mischief when itself is altered?

Look at the plain case. The brain is sound—voluntary motion and sensation are perfect. The brain is altered—sense and voluntary motion are impaired. The brain recovers—sense and voluntary motion are restored. Surely the obvious rationale would be—voluntary motion and sensation depend on the integrity of the brain, and are modified by its alterations. We have just the same evidence of the connexion of the intellect with the cerebrum—of the senses with it. The portion of brain with which the optic nerve is in relation is destroyed—so is sight; or is partially injured—and vision is perverted. Mr. Mayo's reasoning would go to the position, that sight is lost, not because the sense was the result of the right action of the brain, but because the brain transmitted a shock to the optic nerve. We crush a portion of the brain, and the individual loses his memory or becomes an idiot. All believe that this is because memory and intellect depend upon the brain, and are destroyed with its destruction. The evidence is just the same as in the case of hemiplegia. Mr. Mayo's explanation is as applicable to the one as to the other.

Mr. Mayo observes that analogy is for him, and he cites one. Strange that that analogy is evidence against him. The brain, he observes, receives a shock through the medium of the intelligence. The voluntary muscles are affected, and even the heart, confessedly not under the immediate influence of the brain, falters in its action. Supposing this the evidence of shock in one case, its absence should be deemed evidence against shock in another. Now, in the instance of hemiplegia, the heart is confessedly not affected. The patient has lost the use of one side of his body, yet his heart continues beating with nearly the same rhythm as ever. A pretty plain hint that those functions only are interfered with, which the brain has the office of regulating.

The whole reasoning is lax, and many questions are involved that cannot be so summarily settled. Mr. Mayo, for example, asserts, without any qualification, that the action of the heart is not derived from the nervous system. Nay, he esteems this so clear a matter, that it is even placed in the middle of a sentence. If, by nervous system, are merely meant the encephalon and spinal marrow, no doubt the heart is very independent of them; but, if we include also the ganglionic system of nerves, that independence is not quite so clear.

The remainder of the chapter on palsy is occupied with the attempt to answer four distinct questions. We may be sure that those questions are intricate, or they would not be separately treated—that they are important, or they would not be put prominently forward. Let us examine them.

1. *How does it happen that a lesion of one side of the brain invariably produces palsy of the opposite side of the body?*

There is not a student of two years' standing who does not know that this is so. There is not one in ten who can tell why. The explanation is anatomical and so far precise, and the chief admission necessary to give it weight is this, that the nervous influence moves in the direction of the filaments of the nervous matter—an admission which the most resolute stickler for facts may gulp.

"Anatomists therefore look with curious interest to the construction of the enkephalon, in the expectation of discovering some transposition or crossing over of nervous filaments from one side to the other, through which the crossing of the depressing influence, or palsy-shock, may be supposed to be conveyed. After the most careful research, it appears that such a transposition or decussation of nervous threads is to be found in the medulla oblongata alone. Where the medulla oblongata joins the spinal marrow, the *anterior pyramids* throw their fibres downwards, in oblique decussation, each to the opposite side, in such a manner that the right anterior pyramid plunges into the centre of the left half of the spinal marrow, while the fibres of the left anterior pyramid plunge into the right half of the cord."

Mr. Mayo concurs, as most physiologists do, with those who think that these decussating filaments are the channels by which the palsying influence is conveyed from one hemisphere to the opposite side of the body. He does so upon several grounds; first, because these decussating fasciculi are the only ones which have been discovered in the enkephalon; secondly, because the position of these decussating fasciculi is exactly that which experiment and observation lead us to expect to be the place of the transposition of palsy; thirdly, because all the phenomena of hemiplegia, from lesion of the opposite side of the brain, may be explained upon this supposition; fourthly, because even the remarkable cases in which partial hemiplegia of the opposite side is combined with partial palsy on the side of the cerebral lesion, admit of a perfect explanation on the same hypothesis.

The chief facts in its favour are to be found in a paper by Dr. Yelloly. They have been glanced at by Dr. Marshall Hall in the paragraph that we have quoted from him. But they merit more special mention.

1. Sir Astley Cooper divided in a dog the right half of the spinal marrow, at the interval between the occiput and atlas: the dog became palsied *on the injured side*. It may be inferred from this experiment, that the seat of the transmission of palsy is to be found above the atlas.

2. Dr. Yelloly describes a case of hemiplegia, which had affected the *left* side of the body. On dissection, a tumor, of the size of a filbert, was found to have been imbedded in and to have made pressure on the *right* side of the muscular protuberance.

Thus while this case seems to prove that the seat of the transit of palsy is behind or below the pons, the experiment of Sir Astley Cooper establishes that it is above the spinal cord. The intermediate *somewhere* must be in the medulla oblongata or its junction with the spinal cord—*where* the decussation of the nervous filaments has been discovered. The evidence appears to be very satisfactory.

But other phenomena require consideration. The transmission of palsy across the body seems to be accounted for. Will the same explanation equally serve the cases which follow?

"Will," asks Mr. Mayo, "in the first place, the course of the decussating fibres account for the production of numbness, or anæsthesia, as well as of muscular palsy; for the former, although not a constant attendant of the latter, may be combined with it, or even exist without it? Upon this question no one, who has well examined the anatomy of these parts, will entertain a doubt. The decussating fasciculi of the anterior pyramid, on plunging into the opposite column of the spinal marrow, strike into its centre, and implicate themselves nearly as much with the posterior as with the anterior fasciculi; that is to say, with the sentient as well as with the voluntary portions of the cord: so that the wonder is, not that anæsthesia should be produced through their agency, but that it should be so seldom produced, compared with the frequency of simple muscular palsy.

Again: it is evident that palsy of *all* the spinal nerves of the opposite side of the body may be sufficiently accounted for, as a consequence of lesion of the cerebrum, if the anterior pyramids be supposed to transmit the palsy-shock; for the fibres of the pyramids, which are continuous upwards, on the one hand, with the cerebral hemispheres of the same side, or with the seat of lesion on the other hand, are continued downwards into the upper part and centre of that tract, from which *the whole of the spinal nerves* are derived. But how is it possible to account for palsy of the opposite side of the face through the same channel, for palsy of the opposite side of the tongue, and of the opposite facial and auditory nerves? These phenomena may, I think, be thus explained: Where the decussating fasciculi of the anterior pyramid plunge into the opposite half of the spinal marrow, they are implicated, in a wonderful closeness of intertexture, with fibres, which, *in their upward course*, bend towards the places of origin of the ninth and seventh, and of the eighth and fifth of the palsied side. May it not be supposed that this interlacement may be a sufficient means of communicating the palsying influence to the ascending fibres, which are in close relation to the affected cerebral nerves? Thus, the palsy-stroke transmitted to the junction of the spinal cord and medulla oblongata, might spread its influence in either direction separately, or in both together, according to laws which may possibly be hereafter rigorously determined; sometimes striking the body alone with palsy, sometimes the face, sometimes both; sometimes palsying speech, sometimes deglutition, sometimes hearing." 203.

That it *is* by the implication of the fibres of the pyramid with other fibres in connexion with the ninth and seventh is probable. But we feel here the contrast between conjecture and the comparative certainty that the cumulative evidence in the former case afforded us. All is doubt, and we catch at the solution because it is the best, not because it is a good one. Much of the plausibility of Mr. Mayo's argument, owes its force to the employment of language symbolical of actions which probably do not exist. In a mesh of fibres the mind readily seizes the idea of a shock diffused amongst them. But more exact reasoning doubts the fact and disregards the metaphor. Such fact and metaphor are still more unsparingly employed in the passage we proceed to introduce. The reader might suppose that the cerebral influence, was dealing its blows like a hammer in a smithy, and the pith of the argument is the physical, and probably the false analogy.

"But the fifth nerve, why is it so rarely affected in hemiplegia? and the orbital nerves, why do they so frequently escape the palsying influence? These phenomena, it will be evident, are highly consistent with the supposition that the palsying force *strikes* exactly at the point where the decussating fibres of the anterior pyramids terminate. Supposing the paralysing impression *to be received* on this part, its force *upwards* should be weakened in proportion to the distance of each cerebral nerve from that part. But the fifth lies further off than the seventh, the third than the fifth; and something in that proportion is the infrequency of the palsy of these nerves in hemiplegia." 203.

All this is so very and so objectionably vague, that Mr. Mayo will do well to cancel it hereafter. For the present, however, he is not content with a modicum of a hypothesis, but manfully carries it out. The hammer still rings in the theoretical forge, and the physiological smith seems to think that "in for a penny, in for a pound" is a canon in philosophy. For he applies the same description of explanation to the occurrence of hemiplegia from lesion of one hemisphere of the *cerebellum*. The pons consists in great part of filaments which issue from each hemisphere of the cerebellum. These filaments meeting the filaments of the anterior pyramid of the same side, give them a shock, which is communicated to the same part as is paralysed in the cerebral lesion.

To explain the coincidence of hemiplegia on one side, with partial palsy on the other, Mr. Mayo relates a case in which these circumstances occurred. The patient recovered in a great degree; no examination after death could take place. The facts being uncertain, the case explains nothing, and, although Mr. Mayo's conjectures are ingenious, they remain conjectures. We may, therefore, ask our readers and ourselves the second question, with the hope that it may be answered more satisfactorily than the first has been.

2. *What reason can be assigned for the facts, that in general hemiplegia from cerebral lesion, the palsy of the leg is less complete, and capable of being more quickly recovered from, than the palsy of the arm?*

Any body who had heard of the shock which has been eloquently introduced by Mr. Mayo, would at once comprehend the necessity of applying it to the answering of the interrogatory. The distance of the toes from the brain being greater than that of the fingers, the shock is of course transmitted to the former with less violence, and does less mischief when it reaches them. Nothing could be more physically obvious.

But those who doubted the occurrence of a shock before, are not likely to be more satisfied now. If they believed that paralysis was interruption of the influence of the cerebrum, they would conclude that that influence, being most decided in the parts more immediately connected with the cerebrum, its loss would be there more sensibly pronounced. We must remember that, however the encephalon may preside over some of the functions of the spinal marrow, it plays also a part of its own, and its operations are least dependent on the brain the farther it is removed from it.

3. *Why, in a slow attack of palsy, are the muscular weakness and the numbness first felt in the extremity of the affected limb? Why in the hand before the fore-arm—in the fore-arm before the upper arm?*

"In this instance it may be presumed that a diminution of the usual quantity of stimulus or energy, transmitted along the nerves from the organs in which they rise, is the cause of the effect observed. A part of the cord is smitten with an imperfect palsy-stroke; it cannot energize as before, or throw along the nerves which arise from it the usual quantity of nervous force. Upon this supposition it would follow, that the defect of stimulus should first become sensible at the extremity of a limb. The weakened segment of the cord might be expected to be unable to throw out energy enough to fill a long nerve, while it yet might supply with adequate force of stimulation the shorter nerves of the portion of the limb nearer the trunk. The fact presents a remarkable contrast with the last class of facts adverted to, and shews at all events that the two are not referable to one principle. In slow hemiplegia, the arm is struck before the leg; but the hand is struck before the arm." 207.

It is well known that sensations, having their source in the course of a nerve, are frequently referred to its extremity. But more than this. Pressure on a nerve sufficient to occasion some interruption to its functions, or lesion of a nerve, will induce paralysis at its extremity, and its extremity only. We will mention an example of this. A girl had the head of the humerus excised. Abscesses and thickening occurred about the shoulder. Suddenly the *extremity* of the little finger, and of the ulnar side of the ring-finger, became insensible to ordinary stimulants. It was not the whole of those fingers, but their extremities only, that were thus affected. This loss of sensation endured for a long time, but gradually, we believe, subsided. Now here was an instance in which, no doubt, the ulnar nerve was implicated near the shoulder in disease. Yet the paralysis was only felt at its very extremity. This appears to be an instance of the same sort, as the commencement of hemiplegia in the distal end of a limb. Probably the obvious explanation of Mr. Mayo is correct—it is the most plausible one that presents itself. The influence derived from the nervous centre being impaired, that impairment is first felt and most felt at the greatest distance from that centre.

4. *How is it to be accounted for, that muscular palsy is more frequent than anæsthesia ?*

" The reason may be this : The office of the sentient nerves is probably an easier function than that of the motor nerves. In some experiments which I made upon the mode and quickness of reparation of both classes of nerves after their division, I found that the sentient nerves resumed their functions in a shorter time than the voluntary nerves. I divided the facial branches of the seventh and of the fifth nerves on one side in a cat: in the third week sensation had returned, but no sign of returning motion appeared till after the fourth. If this principle be true, it will solve the present question. The sentient nerves, it would appear, require a harder blow to palsy them than the voluntary. It deserves besides to be pointed out, that, as the office of the former is to transmit towards the centre, not from the centre, it is natural to expect that they would be less susceptible of a force proceeding against the habitual course of these motions. The connexion of the decussating fibres of the pyramid with the posterior part of the opposite half of the spinal cord, is likewise not *quite* as extensive as with the anterior." 207.

It is certainly consistent with all that we know, to suppose that mere sensation is a phenomenon requiring less effort in the nervous system, than the propagation of the motor influence. Whether this is the whole or the greater part of the explanation of the phenomenon adverted to, is another matter.

Mr. Mayo concludes the section, by an allusion to the well-known relationship between palsy and apoplexy. The one is, indeed, a greater degree of the other, or, rather, the producing lesions are so related.

One other circumstance deserves notice. It is the occurrence of *convulsions* on the same side as the cerebral lesion, concurrently with hemiplegia on the opposite. A case is related which is very interesting.

*Case.* W. Tucker, ætat. forty-two, brought into the Middlesex Hospital, and supposed to be intoxicated: he was drowsy, heavy, stupid, not insensible, answered some questions ; the pulse small and slow. The left arm and leg powerless, face drawn to right side. When put to bed, he was seized with rigor, and complained of pain in the right side of occiput : in an hour afterwards the pulse rose, and the right side of the body became convulsed: v. s. ℥xviij: the convulsions ceased for a time, then returned with extreme violence, threatening to suffocate him : v. s. ℥xl : the respiration became more free, but the convulsions remained : he then became comatose. He continued insensible during the night, the breathing stertorous, right pupil dilated, left contracted, no pulse at the wrist: he died at 11, a.m.

A large cavity filled with blood, partly clotted, occupied the centre of the right hemisphere of the brain : it did not communicate with the lateral ventricle, but opened between the sulci of the convolutions, which for a large extent were lined with it; between their summits streaks of clotted blood lay, resembling veins.

There was slight sanguineous effusion on the surface of the anterior lobe of the left hemisphere.

It is possible, says Mr. Mayo, but very unlikely, that this may have caused the convulsions of the right side of the body. It is unfortunate that even this degree of obscurity should hang over the case. We lately saw, with an intelligent surgeon, Mr. Skegg, of St. Martin's-place, an instance of hemiplegia upon one side, and convulsions on the other. The patient was a young man, accustomed to drink hard, but of robust constitution. He was suddenly attacked with insensibility and stertor, and hemiplegia of the right side. Soon convulsions of the left side, which was sensible to stimulants, succeeded. No remedies exerted any influence on the symptoms, and, in about thirty-six hours from the commencement of the attack, the patient died.

On dissection, great softening of the left optic thalamus was discovered. It formed in its centre a sort of bouillie, without any appreciable vascular injection around it.

We return to Dr. Hall. We left him at the commencement of our pursuit of hemiplegia. We have agreed that *that* occurs on the side opposite to the one in which the cerebral lesion is found—on the same side as that in which the lesion of the oblong and spinal medulla is situated. He proceeds :—

"It has been further ascertained that, *in experiments,* lesions of the encephalon induce *paralysis only,* whilst lesions of the medulla oblongata and spinalis induce *convulsion* or *paralysis,* according to its severity. Hence it becomes an important question to determine the cause of convulsive affections in *disease* of the encephalon." 34.

Dr. Hall attempts farther on to explain this point. It is well known to surgeons, that a small degree of pressure on the cerebrum will produce convulsion—a greater degree of pressure, paralysis.* If convulsions occur,

---

* Mr. Mayo, in his remarks on apoplexy, goes still farther than this. He observes, and we do not vouch for the correctness of the statement, but decidedly object to its positive character, that it is possible to distinguish when the effusion of blood is on the surface of the brain, because it is then uniformly attended with convulsions. He quotes from Abercrombie the case of a man, a habitual spirit-drinker, who, after drinking whiskey to excess, was found insensible, and had attacks of violent convulsions at short intervals. He died soon after he was thus found, in an attack of the convulsions in question.

On removing the skull-cap after death, an appearance was observed on the surface of the dura mater, of coagulated blood in small detached portions.—These appeared to have been discharged from small glandular-looking elevations on the outer surface of the dura mater, which were very vascular, and highly gorged with blood. There were depressions on the inner surface of the bone, which corresponded with these bodies. On raising the dura mater, there came into view a coagulum of blood, covering and completely concealing the right hemisphere of the brain ; it was about two lines in thickness over the middle lobe, and became gradually thinner as it spread over the anterior and posterior lobes, and dipped down below the base of the brain. The coagulum being removed, measured about ℥v. On the surface of the left hemisphere, the veins were turgid with blood ; on the surface of the right, they were entirely empty ; but the source of the hemorrhage could not be discovered. There was no fluid in the ventricles, and no other disease was discovered.

as they occasionally do, after an injury of the head, that is presumptive evidence of moderate pressure. To us there appears no greater difficulty in accounting for convulsion from cerebral lesion, than in accounting for hemiplegia. If it is demonstrable, which it is, that a small quantity of blood effused upon the brain will give rise to convulsion, it is not probable that, in *experiments*, lesions of the encephalon *do* produce paralysis only. What is the effusion of a little blood but an experiment? It matters, we suppose, little, whether that be effected by an intentional or an unintentional blow from a bludgeon. The effect is the same in either case—a little blood is extravasated. If convulsion follows in the one instance, which it does, why should it not follow in the other, which Dr. Hall assures us it does not? We would recommend him to re-consider this passage. But how is convulsion from cerebral disease or injury to be explained? Dr. Hall offers the following solution, which, we must own, does not, to our apprehension, do much more than express the actual fact.

"Disease of the meninges and of the brain, induces spasmodic actions. How is this explained? I think upon the principles of irritation and counter-pressure. The first *may* act through the medium of the nerves distributed to the membranes,—as the recurrent of the fifth of Arnold. In reference to the second, I may adduce several valuable facts. In an interesting case most anxiously watched, and accurately detailed to me, by my friend Mr. Toogood, of Bridgewater, of a little girl, aged thirteen months, the croup-like convulsion occurred repeatedly, until one day, when the bones of the cranium separated, the convulsion then ceased; in a case of spina-bifida related to me by Mr. Herbert Evans, of Hampstead, there was a croup-like convulsion whenever the little patient turned, so as to press upon the tumor. In the case of anencephalous fœtus, described by Mr. Lawrence, convulsion was produced on pressing on the medulla oblongata. In a case of meningitis given by Dr. Abercrombie, the anterior fontanelle became very prominent; pressure upon it induced convulsion. Hypertrophy of the brain affords an argument of the same kind: it induces convulsion, except in the case in which the cranium grows with the encephalon. These and other facts lead me to think that convulsion arising from cerebral disease, is thus to be explained." 44.

The only explanation that we discern is contained in this sentence—"upon the principle of irritation and counter-pressure." We may be obtuse, but really we conceive that explanation to amount to very little, and to leave a great deal to the genius of the reader. We repeat that there is no more difficulty in explaining the occurrence of convulsion than of hemiplegia. The only apparent difficulty is in the inconsistency of the former, following disease of the encephalon, and not experimental lesion—an inconsistency, which, for the reasons we have stated, we do not believe to be real. Unfortunately, however, the numerous experiments that have been made upon the brain have not dissipated the uncertainty that hangs over its precise functions. On the contrary, they have, in many instances, tended to render the obscurum obscurius, and, multiplying contradictory facts, have only thrown doubt on what was formerly deemed certain. That this is not an exaggerated view of the present state of the physiological pathology of the encephalon will be evident from the following passage.

"I must now," says Dr. Hall, "briefly state to you that, formerly, Saucerotte, in his Prize Memoir presented to the Académie Royal de Chirurgie, in 1768; and, more recently, MM. Foville and Pinel-Grandchamp, M. Serres,

M. Lacrampe-Loustau, and M. Bouillaud, have attempted to shew, that, besides this crossed effect of the cerebrum, affections of the corpus striatum, or its *middle lobe,* induce paralysis of the *inferior* extremities ; whilst similar affections of the thalamus, or its *posterior lobe,* induce paralysis of the *superior* extremities ; so that, if this opinion were true, there would be a *doubly crossed effect.* I use this phrase as a sort of *mnemonic* for you, if you should wish to speak of these opinions, for I fear I must call them by that name: M. Lallemand and M. Andral, after an examination of an extensive series of facts, have declared that the statement is without foundation. M. Bouillaud has further attempted to shew, that disease, or lesion, of the *anterior lobe* of the cerebrum leads to a loss of the power of articulation. But this opinion is equally contested by the two authors whom I have just quoted.

I must now briefly notice an attempt to *localize* the affections of the brain of a different kind, but equally disputed by these pathologists: MM. Delaye and Foville have stated that the grey or cortical substance is principally affected in *mania ;* MM. Bouchet and Cazauvieilh, whilst they agree with MM. Delaye and Foville in their view of the pathology of mania, contend that in *epilepsy* it is, on the contrary, the white or *medullary* portion of the brain which is diseased.

The tubercula quadrigemina alone have a crossed effect, both of convulsion, and paralysis.

M. Ollivier observes that a hæmorrhagy into the tuber annulare only paralyzes the movements; M. Cruveilhier, on the contrary, asserts that such an affection destroys the sensations and the movements, but leaves the intellect uninjured. How many questions, then, still remain for future inquiry to solve!" 35.

How many indeed.

Dr. Hall adds, what is pretty well known, that in those cases in which hæmorrhagy occupies an extensive space, affecting both hemispheres of the cerebrum,—as in meningeal hæmorrhagy at the summit, or at the base of the brain, in extensive hæmorrhagy within the brain, extending from one hemisphere to the other, or into both ventricles,—*general* paralysis is observed; the same event takes place in the cases in which a clot is formed in the mesial line in the tuber annulare.

We stride over many pages to notice other kinds of paralysis. Their connexion with the brain is less direct, or more obscure than that of the forms of palsy we have quitted. To the practitioner and the physiologist they are interesting alike.

1. The first is Paralysis from Dental Irritation. Dr. Hall gives a case as a description.

*Case.* The child of Mr. Grant, an intelligent practitioner of Thayer Street, was twenty months old, and had been suffering for some time from dentition, being fretful, and having a cough during the night. On the morning of April 30th, 1835, her mother observed that she was incapable of raising the right arm; she retained the power of swinging the arm backwards and forwards, and of bending the forearm on the arm, but had not the least power to raise the arm itself, as if the deltoid muscle only was paralyzed. On examining the arm, the child suffers no pain, and there is not the least reason to believe that any accident could have occasioned this loss of power. The general health of the child, with the exception above-mentioned, is excellent; appetite good; bowels are every day relieved.

Dr. Marshall Hall, on seeing the child, recommended a gentle emetic, followed by a dose of castor oil; the gums over the four eye-teeth, which are all coming forwards, to be carefully lanced every second day; and, ultimately, an embrocation to the arm; and a light unirritating diet.

Thus the child went on with little alteration till the evening of May 6th, when she had several fits of coughing, resembling the convulsive crowing of croup. There was no fever, and nothing was done till next day, when the pain was removed from the back of the head, two leeches were applied behind the ear, and the other remedies were persevered with. On the 14th, the child had evidently regained some power in raising the arm, and there had been no return of the crowing cough.

On the 21st, one of the teeth had arrived at the surface, and the others were advancing. On the 10th of June, she had nearly recovered the complete power of her arm, and her night-cough was almost gone. The other teeth were not quite through the gum. The child got perfectly well. It is scarcely necessary to repeat that the treatment consisted in lancing the gums —opening the bowels—and giving light diet.

2. "We frequently observe," says Dr. Hall, "a *hemiplegic* paralysis from defective development of the opposite hemisphere of the cerebrum. In this case, *both arm* and *leg*, but chiefly the arm, are involved in the paralysis. But it occasionally happens that *one leg only* is affected with a *partial* paralysis; the limb does not *grow* as the other leg does, but remains thinner and shorter; yet it does grow, so that the paralysis is not complete; and it is moved, only with somewhat less power than the other leg.

What is the nature of this partial paralysis? Is it of dental origin? Is it an affection of the spinal marrow, or of its nerves, equally partial? Cases and careful examinations are entirely wanting to determine these questions." 84.

Dr. Hall cites a very interesting case in the words of Dr. Webster, of Dulwich, the father of the child.

"When my boy," writes Dr. Webster, "was about twenty-months old, (he is now nine years,) he had a fit of illness, connected with dentition, which threatened the brain, and for which I opened the jugular vein and purged him. This took him off his feet; and, very soon after, he had a fall from a rather high cribbed; but this was not attended or followed by any apparent bad consequences. The child recovered his health; but, for some weeks, he seemed to have almost entirely lost the use of his legs, and, being uneasy about him, several of my medical friends saw him; amongst others, I think, yourself. He gradually, however, began to walk again, but not so steadily as before, as he tottered much in his steps, and was constantly falling over every little object that happened to be in his way, and he had much less command over the left limb than the right. He seemed to walk on his toes. It was not at first ascertained that one leg was more affected than the other; but as he grew up and was breeched, the matter became more apparent; he plainly walked more firmly on the healthy limb, and less so on the lame one, and he threw it about more in walking and playing, and rarely set down the heel, except when walking slowly, never when running. He now runs on the toes of that foot, and with a sort of lurch; the limb is less firm, the muscular power is evidently less, but the sensibility seems equal to the other.

I have only to add, that the affected limb is about an inch shorter than the other, which is the reason of his walking on the toes." 85.

In juxta-position with this case is one taken from Dr. Abercrombie's work, which Dr. Hall may well style able. A very admirable and philosophical work—a treasure of facts to all who shall come after. We cannot refrain from introducing it, in order that our notice of this matter may be more complete.

"It is now upwards of twenty years since I first saw a girl, aged, at that time, about eighteen months, and previously enjoying excellent health. She had been left for some time sitting upon damp grass, and was immediately seized with fever, accompanied by such a degree of oppression as led to an apprehension of an affection of the brain. These symptoms, however, passed off in a few days, and, upon her recovery from them, it was found that she was entirely paralytic in the right lower extremity. She has, from that time, enjoyed uninterrupted health, and is now a tall and strong young woman; but the right lower extremity has continued entirely paralytic. It is also a great deal smaller than the opposite extremity, and several inches shorter. All the joints are remarkably relaxed, and the muscles flaccid; but there is no other appearance of disease in any part of it, or in the spine." 85.

### Apoplexy.

From palsy we turn to apoplexy, two affections allied in cause, and commonly co-existent in fact. We shall not, of course, give a history of apoplexy, but merely select what has something in it to attract our special notice.

It is scarcely necessary for us to mention that from the slighter shades and symptoms of cerebral oppression up to what is familiarly called apoplexy, there is no natural resting place. The derivation of the term implies no more than suddenness of seizure, and a philosophical consideration of the disease does not tend to confine definition within narrow bounds.

Practically speaking, there is no point connected with apoplexy so important, as recognizing the slighter forms or the premonitory symptoms of it. Dr. Hall properly insists on this, and it is indeed of primary consequence. As we know that apoplexy is rather a disease of middle and advanced than of early life, it is at the former periods that we watch most anxiously for those seemingly trivial indications of cerebral oppression, which to the well-informed medical man are so significant.

The morbid anatomy of apoplexy is far from affording such decisive data for diagnosis and treatment as might be wished, and, indeed, expected. We find—rupture of vessels and extravasation of blood—serous effusion into the ventricles or on the surface of the brain—congestion of its venous system, without rupture—and softening of the brain—and we find nothing at all.

Nor is severity of symptoms proportioned to extent of lesion. In many severe cases, the morbid appearances may be very slight, or there may be none—and in cases, in which the symptoms are not severe, the lesion may be most extensive. Mr. Mayo quotes from Dr. Abercrombie a case which exhibits the absence of any absolute ratio between organic alteration and symptoms, and we dwell on this point the more because, in practice, astonishment is frequently expressed by medical practitioners, when a statement of the sort is made.

*Case.* A man, aged fifty-four, of a plethoric habit and short-necked, was admitted into the clinical ward of the Edinburgh Infirmary under the care of Dr. Duncan on the 31st of May. He was in a state of nearly perfect coma, speechless, and with palsy of the right side to such an extent that even the intercostal muscles of that side did not act. The leg and arm of the left side were occasionally affected with convulsive motions. Breathing stertorous, deglutition much impaired, pulse 74. The affection was of three days' standing, and had come on with vertigo, loss of vision, violent headach, and vomiting. The usual remedies were productive of no service. On the 1st of June there seemed to be a slight return of intelligence, but coma soon recurred, and the patient died on the 3rd.

*Dissection.* No farther appearance of disease could be discovered, than that the choroid plexus seemed rather darker than usual, and the basilar artery was diseased at one spot. By the side of the artery, there was a spot of the cerebral substance, no larger than a barley-corn, which appeared somewhat softened, but even this Dr. Duncan considered as extremely doubtful.

Who would not have said that in such a case, at such an age, organic lesion would surely have been found?

The evidence on which we conclude that pressure, generally from blood, is the essential cause of apoplexy, seems to be very clear and satisfactory. That evidence commences, perhaps, with such a case as the one related by Zitzilius; where, a boy had drawn his neckcloth remarkably tight, and was whipping his top, stooping and rising alternately, when after a short time he fell down apoplectic. The neckcloth being unloosed, and blood being drawn from the jugular vein, he speedily recovered. Or perhaps it might start with the experiment of Sir Astley Cooper—who trephined a dog, and applied some pressure with his thumb upon the dura mater. The dog instantly became insensible, and had the symptoms of apoplexy. Sir Astley removed his thumb from the dura mater, and gradually the animal's sensibility returned. This appears decisive, and from this experimental pressure up to the case of compression from depressed bone, or from effused blood, scarcely a link would seem wanting in the chain.

Yet what point is there in medicine so settled, as not to foster doubt and admit of disputation? It has been, and it is a question with many, whether pressure is the essential physical cause of apoplexy. Mr. Mayo puts that question fairly:—

"The cases, which have been detailed, if they exemplify the features of apoplexy, exemplify no less the extreme difficulty of assigning their physical cause. In cases of sanguineous, or serous effusion, it appears natural to consider the mechanical pressure as the cause of stupor. But the stupor is often slow in coming on, and often does not manifest itself till some time after the effusion has taken place; nor is it necessarily permanent, although the effusion remains: for it may be temporarily relieved by bleeding, or without any apparent cause gradually wears off. And, if it would not encumber this volume by too many illustrations, cases might be adduced, in which large extravasations of blood have existed without any degree of coma; and still more numerous and striking cases of enormous accumulation of water in the heads of adults, without a single apoplectic symptom. The extravasation, or pressure of effused fluid, where it exists in apoplexy, is probably therefore a part only of the physical cause of coma; something there must be in addition, some change either in the condition of the nervous substance, or in the cerebral circulation, that is brought on by, although, as in the first instance quoted, capable of existing independently of, any effusion, to constitute the immediate cause of the mental oppression.

The symptoms of apoplexy bear, if not a certain, at all events the most definite relation to the organic lesion, when they flow from sudden extravasation of blood." 187.

In order to set the operation of pressure in a proper point of view, it is necessary to attach due importance to two circumstances:—1. The suddenness of the compression—2. The possibility of pressure having existed during life, when no certain proof of it continues after death.

The evidence of pressure is positive. For, the thumb is laid on the dura mater—or the bone is knocked in with a bludgeon—or blood is suddenly effused within the cranium, and instantly apoplexy follows. If those causes are removed, the apoplexy, in a fair case, where too much mischief *has not been* inflicted, is removed too.

Suddenness of compression is shewn by conclusive evidence to be an essential element in the production of decisive symptoms. No fact can be better made out, than the symptoms of pressure on certain parts of the spinal marrow, or on the medulla oblongata. Yet in cases where the compressing force has acted slowly, the most extraordinary lesions have been unattended with specific symptoms.

We are not to conclude, because we find no blood effused, or no palpable congestion after death, that therefore no congestion has existed during life. The case of Zitzilius, the cases that every day present themselves, shew how fleeting cerebral congestion, attended with dangerous symptoms, may be. Such pressure may have existed to a degree fatal to the performance of the functions of some brains, yet in death or after death, when the circulation is so modified, it may have ceased to be apparent.

The evidence against pressure is not positive. It chiefly resolves itself into the absence of the proof of pressure after death. But this, as we have observed, is inconclusive, for it can be shewn that pressure existed and did injury, when after death, it has disappeared, and the laws of the cerebral circulation will explain many of those phenomena, which have been thought to tell against the theory of compression. No doubt cases may be picked out, where what must occasion pressure did not occasion apoplexy. But there is no instance in which the exception less destroys the rule, than this of the brain. There are no facts respecting it, decisive as they may seem, which have not striking exceptions. If any thing seems clear, it is that sudden depression of a portion of the cranium will produce the symptoms of compression; but it is *as* clear that such depression has occurred and persisted without occasioning such symptoms. It is hardly fair to say, that this disproves the operation of pressure, for it equally disproves the operation of every thing. It not only tells against any theory of consequences, but it tells against the *fact* of consequences, which is an absurdity. The true inference from such cases is, that we are not sufficiently acquainted with all the conditions of the cerebrum, nor with the laws of its functions, to be able to appreciate the disturbing circumstances in a given case. On the whole, we think the arguments are in favour of the physical action of compression constituting the most essential element, in the production of what we call apoplexy.

It might be expected that pressure about the cerebellum, or at all events, in the vicinity of the medulla oblongata, would be more rapidly fatal, than on the surface or in the substance of the cerebrum. Much of the latter is probably devoted to the performance of functions intellectual, rather than merely vital. The fact, at all events, is as we have stated.

### ACCIDENTAL APOPLECTIC SYMPTOMS.

There are some symptoms which may, perhaps, receive without much impropriety this designation. Sometimes they are present—sometimes they are not—and we know not, in many instances, why they should either exist or be absent. Practically speaking it is important to observe them.

Vertigo is such a symptom—Wepfer mentions the case of a woman, who was recovered after hanging, by frequent bleeding; she was for some time afterwards affected with vertigo, which subsided gradually.

Vomiting is another symptom of this sort. Dr. James Johnson attended a female servant whose chief symptom was obstinate vomiting. He sent her to St. George's Hospital, where, after ineffectual treatment, she died. No disease was discovered in the stomach, but an abscess existed in the cerebellum.

We were lately consulted on the case of a little boy who had been under the care of several eminent physicians. His chief symptom was vomiting, with a little pyrexia. We could discover no obvious lesion of the stomach. But the boy was of a lymphatic and strumous habit, and appeared rather heavy and drowsy. We determined to treat the case for a head affection, fearing there might be scrofulous tubercle in the cranium, or some organic mischief going on in it. We ordered repeated leechings on the temples, succeeded by blisters behind the ears, and aperient medicines. In a few weeks the boy had quite recovered, although he had been previously leeched and blistered very liberally on the stomach to no purpose.

"The alliance between vertigo and sickness is remarkable. In injuries and diseases of the head, these feelings perpetually accompany each other; and there are curious instances which even go to show that the one has a necessary connection with the other. In persons sick at sea, it is very certain that the shifting motion of visible objects, and the instability and want of support felt, or, in other words, the disturbance of the sense of equilibrium, and the giddiness so produced, are the causes of the sea sickness. If a person on ship-board, with feelings of nausea coming on, lie down and close his eyes, the nausea is wonderfully diminished; yet the only difference which he has made, is excluding impressions which favour the production of giddiness. When a person has drank immoderately of wine, on the contrary, if he shuts his eyes he experiences a strong vertigo or sense of whirling, followed by nausea; but if, instead of giving way to this sensation, he fixes his sight on a stationary object near him, the vertigo is corrected by the steadiness of the visible object; and as the vertigo disappears, the nausea wears off. The mitigation of the nausea is in both of these dissimilar instances the effect of getting rid of the vertigo." 194.

Headache is another symptom of the kind that we have been speaking of. Headache has many causes. We allude only to one form of it, happening under certain circumstances. Tissot for instance, so Mr. Mayo writes, mentions a woman, who, after complaining for some time of headache, was attacked with a great and sudden increase of pain, accompanied by loss of sleep, and died in a short time. On dissection no morbid appearance could be detected. A young woman, mentioned by the same writer, having, during the flow of the menses, suffered from a fright, the discharge stopped, and she became liable to frequent leipothymia. After suffering from this and various other symptoms for several months, she fell into a profound sleep, from which nothing could rouse her; this continued four days; she then came out of it and appeared to be recovering, when, after several days, she was seized with severe headache, anxiety, and convulsions, and died. No morbid appearance could be detected in any of the viscera.

We quite agree with Mr. Mayo that the pathology of such headache is analogous to that of simple apoplexy. The affections are allied. There is no doubt that, exhaustion and extreme depletion will occasion headache. We cannot wonder at that, when we recollect what the effect of both is on the heart. This becomes irritable, and acts with rapidity and violence. What blood there is, is driven with force through the organs. We may presume that the effect on the head is headache—we know that in the arteries we have the bruit-de-soufflet, the indication of the extreme action excited by the heart. We remember the case of a man who had been furiously bled for hepatitis. He had violent headache, and throbbing in the cranium, and the bruit-de-soufflet was distinct in every artery of any size. He died and no lesion was found either in the head or heart.

In private practice we not unfrequently meet with such cases as the one we shall quote from Mr. Mayo. They are often perplexing, and the more so because the cautious physician reflects that a little slip on his part may be disastrous in its consequences. On the whole it is better to lean to the side of depletion: an error on either side soon renders the case more obvious. But it is better that a man should be obviously weak, than that he should be obviously apoplectic.

"A gentleman," as our author tells us, "between his thirty-fifth and fortieth years, of a temperament inclining to nervous, but stout and muscular, habitually taking considerable exercise, and living heartily, and engaged in an active profession, has experienced, on different occasions, when exhausted by days of previous fatigue, the following attacks. After partaking early of a hearty breakfast, and travelling twenty miles, he was seized with vertigo, and inability to stand, and slight nausea: he took two glasses of brandy without any effect; but became relieved upon producing vomiting, by means of salt and water, and lying perfectly still. On another occasion he awoke in the night, low, depressed, shivering violently, with a sense of failing power, like apparent dissolution; this was relieved by applying hot bottles to the feet, and administering brandy. On another occasion, when exhausted by over-exertion, he was taken with a sense of feebleness joined to imperfect vision; in which, at first, he could see only the objects to which the optic axes were immediately directed; in half an hour afterwards, this was changed to vision of the half of objects: with quiet and stimulants, in another half hour these sensations wore off. These seizures were followed by slight headach. He is now in perfect health; but feels on going through much excitement or exertion, that he might bring on a return of the feelings described." 194.

We quit the consideration of apoplexy, to glance at almost similar pathological states—those of concussion and compression. It is impossible for any surgeon to understand or to treat either well, who is not familiarized with the phenomena of those affections of the brain which usually fall under the care of the physician. The latter would acquire an insufficient idea of the complaints *he* ministers to, were he not aware of the consequences of injuries of the cranium. Let us, then, glance at—

CONCUSSION AND COMPRESSION.

1. The direct effects of concussion, or what is in common language called "concussion," are stated by Mr. Mayo to be, "syncope and something more." What the something more is, it is easier to imagine than to say, for, in truth, the exact definition of "concussion" is difficult. In some surgical works in the hands of students, no such difficulty would appear to be experienced; the positive symptoms and precise diagnosis of "concussion" and "compression," are there laid down, and they even go so far as to determine the opposite condition of the pupil in each.

It is interesting to inquire, but difficult to ascertain, what is the alteration that the structure of the brain undergoes in concussion. The opportunities of examining the bodies of persons who die from simple concussion are rare. It appears that it is, in some cases, impossible to distinguish any structural lesion—while, in others, laceration of the substance of the brain and ecchymosis may be found. Ecchymosis is only extravasation on a small scale, and, when it is present, the case trembles on the confines of concussion and compression. The symptoms of either are not separated by a broad and distinct line of demarcation, but are insensibly blended and confounded.

The occasional consequences of concussion have attracted curiosity. The operations of the intellect are interfered with, and memory particularly suffers. Dupuytren relates the case of a person, who recollected only the syllables pa pa. This small stock of language was all that was left to him. The cases published by Sir Astley Cooper and Sir B. Brodie are notorious.

Mr. Mayo points out the analogy between concussion and compression; —The instance, he says, in medical pathology to which it is most parallel, is the first stage of apoplexy from rupture of a vessel, which is characterized by paleness, feebleness of the pulse, and vomiting. In that case, in truth, the cause of the symptoms is equally mechanical—a blow from the gush of blood upon the brain. No doubt that, in each instance, the cerebrum receives a shock.

2. Compression of the brain from mechanical causes, does not differ in symptoms from apoplexy with extravasation. The study of one elucidates, and is necessary for the appreciation of the other. Whether the compression be from bone, or blood, or matter, does not signify, quoad the compression. But, in the latter case, inflammatory symptoms are associated with those of pressure, and may modify them. The following is an interesting case of the effects of suppuration.

"A young man had a small portion of the left parietal bone denuded by a blow; it became necrosed, and was already beginning to loosen, when he observed that the thumb and fore-finger of the right hand were weak and numb: in a day or two he was taken with a slight epileptic seizure, which lasted four or five minutes: a similar fit recurred a few days afterwards. This I attributed to the confinement of matter between the bone and dura mater; but as the bone was loosening, it was evident that there would shortly be a free escape for the matter; so that I thought it better not to apply the trephine unless the symptoms became more urgent. As the ulcerated groove around the dead portion of bone enlarged, the palsy of the thumb went away, the patient had no return of the epileptic seizure, the dead bone separated, and he recovered." 224.

Unfortunately, it seldom happens that when matter forms upon the dura mater, after an injury of the head, an operation is sufficiently early to prevent extensive and fatal mischief. We have seen the skull trephined often when symptoms of suppuration in the cranium had occurred, but in no one instance has that operation been in such a case successful. On dissection, we usually discover that the suppuration on the surface of the dura mater is not limited to one spot, and frequently there is suppuration *under* the dura mater, and inflammatory softening of the brain in that situation. Yet favourable cases do now and then occur, to teach the surgeon not to abandon hope entirely. For instance:—

Sophia Pennett, æt. sixteen, was admitted into the Middlesex Hospital under the care of Mr. Joberns, on the 19th of October, 1815. She had been knocked down in the street by a carriage, the wheel of which had grazed the left temple and denuded the parietal bone. She had lost a considerable quantity of blood from the temporal artery, was low and weak, and vomited. The part exposed did not recover: about a third of the surface of the parietal bone was necrosed. November 9th, having gone on favourably in the interval, but complaining occasionally of pain in the back of the head, she was taken with shivering and sickness. Nov. 10, she could not articulate: pulse 90, and irregular; at times insensible. 12th, cannot put out her tongue: right side of the face and arm paralytic. Nov. 14th, Mr. Joberns trephined the parietal bone, when about a teaspoonful of matter escaped, which had been confined between the bone and dura mater: the latter was covered with a layer of organized lymph. The operation was performed about one o'clock; it was followed by an immediate amendment. Nov. 15: now quite sensible; her speech in great measure returned; she can move her arm. From this period her recovery was rapid.

A very fortunate case.

Blood may be effused *under* the dura mater. Cases have occurred, where, on trephining, the dura mater was raised and rendered blueish by blood beneath it, and when the prominent membrane has been punctured, the blood has spirted out to the height of seven feet. Sir B. Brodie has put on record a very remarkable fact of this description.

The following is a short summary offered by Mr. Mayo, of the different effects of compression.

"1. Coma without stertor: commonly resulting from extensive cerebral laceration and hemorrhage; but sometimes from remediable extravasation.

2. Coma with stertor, and partial hemiplegia; often resulting from depressed bone, or circumscribed extravasation, or suppuration, upon the dura mater.

3. Coma with violent convulsions; resulting from extravasation on the surface of the brain, sometimes situated without the dura mater.

4. Epileptic seizures; from small circumscribed effusion [or depression of bone ?] upon the dura mater.

5. Acute pain in the head, from depression of bone.

6. Sudden and great decline of frequency in the pulse. This I witnessed in a child, supervening several days after fracture of the skull. The symptom was followed in a few hours by coma and hemiplegia: there was extensive suppuration between the bone and dura mater." 228.

The catalogue is not quite complete, for other consequences from compression have been witnessed.

7. Delirium is a symptom that sometimes results from pressure on the brain. We will relate a case which fell under our own care.

*Case.* A man was admitted into St. George's Hospital, after having fallen on his head. He was stunned for about ten minutes, but soon recovered his sensibility. Yet the sensibility was not perfect. When left to himself, he would continue talking in an incoherent way, and, when spoken to, said he would give us " a punch in the head." When asked if he had a wife, he replied—" Yes, too many." We should observe that there was extensive ecchymosis in the frontal region, and particularly in the neighbourhood of the left eye. On the third or fourth day,* he was suddenly seized with what looked like delirium tremens, but the delirium soon assumed a very violent character, and it was necessary not only to put on a strait-waistcoat, but to secure him to the bed, and set powerful men to hold him down. It was the most furious delirium we ever saw. What was curious, it was of an erotic character, and he used the most obscene language, and invited the nurse to go to bed with him. After this had continued for some hours, he gradually sank into the state of coma, with stertor, and died.

On dissection, it was found that the frontal bone was extensively fractured, the fracture running across the æthmoid, into the sella furcica, and both temporal fossæ. Between the bone and the dura mater, opposite the anterior part of each cerebral hemisphere, was a large quantity of firm black coagulum. Opposite the cribriform plate of the æthmoid bone, the dura mater was lacerated, and the laceration extended into the substance of the brain, which contained about two drachms of blood, and seemed slightly softened round it.

In this case, the symptoms were at first those of concussion. Afterwards they certainly were not the ordinary ones of compression. Yet there was an extensive fracture, with laceration of the dura mater and of the brain, blood *on* the former, and *in* the latter. From first to last there was a disposition to delirium. At first it was so slight as to be considered the result of intoxication—afterwards it was very furious, and lapsed into apoplectic insensibility. There was no appearance, however, in the lesion, of change corresponding to these great variations in symptoms. Could the slight softening round the clot in the brain have modified the latter symptoms? We see the same round apoplectic clots, without delirium.

This case occurred six years ago, and we saw none similar to it until the year before last. A woman was then admitted into St. George's, after having received a blow upon the head. She was insensible at the time, but recovered her consciousness in a great degree. On the second or third day she became delirious, furiously delirious. There was no heat of skin, the pulse was not accelerated, no symptom of inflammatory action was present. Recollecting the preceding case, we expressed our suspicion of pressure from extravasated blood, there being no evidence of fracture. In this opinion no one acquiesced. The woman died. On dissection, there was found extensive effusion of blood on the dura mater, beneath the left parietal bone, and no other lesion could be found.

Delirium, then, must be received as an occasional effect of compression.

---

* We have full notes of this and of another similar case, but we cannot at this moment refer to them.

The only evidence of its cause during life, with which we are acquainted, is its sequence to an injury of the head, and its not being accompanied with the signs of inflammatory action.

8. In one case we saw extraordinary bulimia after an injury of the head, attended with all the symptoms of compression. The case was that of a boy. He would steal everything eatable in the wards that he could procure. The bulimia gradually subsided. Perhaps this was only a post hoc. We mention the fact, but attach little importance to it.

9. Mr. Mayo makes no mention of *perverted* sensations and *perverted* intellectual operations, as a consequence of compression. He mentions only coma and hemiplegia. Yet we all know that, after compression, there have been instances in which particular senses have been altered. One patient always perceived a foul smell, and things that formerly gave a pleasant odour now seemed to emit a stench. Loss of memory, particularly in regard to special objects of it, has been noticed. We need not multiply examples of a fact which has been frequently noticed and recorded.

10. If we recollect rightly, the loss of the power of properly generating or supporting animal heat, has been observed after an injury of the head. Mr. Guthrie, we believe, has published the case of an officer, one side of whose body is in this predicament. It is so much less able to support its temperature than the other, that the gentleman is obliged to wear on it a vast quantity of additional clothing.

### Epilepsy.

On this head Mr. Mayo's observations are so meagre, that we leave him and turn to Dr. Marshall Hall.

He draws the distinction, or rather he insists on it, between *centric* and *eccentric* epilepsy. Centric epilepsy has its origin in lesion of the brain or spinal marrow*—eccentric epilepsy has its source in irritation of the nerves. The terms are new—the practical application of the view is old. All have been aware, that epilepsy may depend on organic lesion of the centre of the nervous system—or on irritation propagated to it from without. The difficulty has been to decide, in a *given* case, which is the disease. In this, Dr. Hall does not help us, nor will any speculative view assist us. The essential elements of diagnosis are—first, the knowledge of the distinction, which is old and general; secondly, the investigation of symptoms in the particular instance.

It is in the nervous system particularly, that we perceive distinctly how morbid anatomy forms but a portion of pathology. The functions of the

---

* We write, in lesion of the brain *or* spinal marrow. Dr. Hall adopts the aphoristic style, and should, therefore, be concise and clear. Concise, indeed, he is, but frequently not clear. So far as we see, he regards epilepsy as a centric disease of the spinal marrow, when centric—as an affection of the nerves when eccentric. But, disregarding theory, there are numerous cases in which mechanical irritation of the surface of the *cerebrum* occasions epilepsy. Surely this is centric epilepsy, and centric *quoad* the cerebrum, and not quoad the spinal marrow. Dr. Hall indeed, points out encephalic tumors, hypertrophy, &c. as causes of epilepsy.

system are so subtle, and so essentially connected with every physical and psychical act, with every function of the organization, and with every part of it—the operations of the nervous centres are so dependent on the impressions conveyed to them from nerves, themselves are exposed to such numerous sources of disorder, that we cannot wonder if this system, above all others, displays a long series of functional affections. How true is the remark of Andral, which Dr. Hall has quoted—how calculated, coming from a pathologist of his character, to put to shame the small fry of arrogant morbid anatomists, who, for every why in symptoms have a wherefore in organic lesion.

"Que le nombre d'altérations connues," says M. Andral, "est petit à côté de celui des lesions qu'on ignore! Les cas où, après la mort, un trouve quelque chose d'appréciable pour le scalpel sont les plus commune pour les autres organes; pour le système nerveux, c'est tout l'opposé: les cas ou on rencontre des lésions sont de beaucoup les plus rares. Cette assertion paraîtra paradoxale à ceux qui ne connaissent des lésions nerveuses que les trois ou quatre maladies qu'on observe dans les hôpitaux; mais les affections nerveuses se comptent par centaines, et pour ne parler que de ces grandes perturbations qui portent sur le mouvement, sur la sensibilité, sur l'intelligence, où est la lésion dans ces cas? La plupart du temps on n'en trouve aucune, ou celles qu'on observe n'ont aucun rapport avec les désordres fonctionels." 89.

Dr. Hall applies the observation to the eccentric diseases of the true spinal system. But it may be applied also to some of the affections of the cerebrum. How uncertain is the morbid anatomy of mania. Dr. Hall's own observation on this point is equally sarcastic and correct.

"The evidence from the morbid anatomy is quite deficient for practical purposes, unless we are enabled thus to distinguish cause and effect; and I fear this point has not been sufficiently considered by those who have addicted themselves to this department of medical science. It is sad to observe how a little effusion, a slight layer of lymph, is the *cause* of every thing, in the minds of some of these gentlemen of one idea." 107.

So difficult is it for the human mind to avoid extreme opinions and conclusions, that the majority of those who do not overrate morbid anatomy underrate it, and, opposed to the gentleman of one idea is the gentleman of none.

Centric epilepsy, whether it depend on lesion of the cerebrum or of the spinal marrow, is in the majority of instances beyond our reach. Eccentric epilepsy is remediable in correspondence to the actual condition of the part, irritation of the nerves of which produces it. We pause on this matter, for it is important, and, Dr. Hall's remarks have a philosophical character.

"The eccentric epilepsy is to be viewed as *curable*, however *difficult* of cure. By avoiding the exciting causes, its attacks are avoided, the susceptibility to returns subsides; these returns become less frequent, and less severe, and at length frequently cease altogether. Every thing depends upon rigid rules proposed by the physician, and strictly and perseveringly observed by the patient." 143.

The causes that he points out, are, 1, the presence of indigestible food in the *stomach*; 2, the presence of morbid matters in the *intestines*; 3, *uterine* irritation. But we may add to these, injuries of nerves, and organic alterations of the nerves, either from injury, or from alterations of the parts about them. Such eccentric epilepsy will be curable or incurable according to circumstances.

Dr. Hall's analysis of the symptoms is at once precise and elegant.

"The first thing observed is a varied *distortion of the eye-ball*, which is drawn from the axis of vision, generally upwards, and outwards, or inwards.

The second symptoms are a forcible *closure of the larynx*, and *expiratory efforts*, which suffuse the countenance, and probably congest the brain, with venous blood. In all these circumstances there is a most marked and important difference between epilepsy and hysteria, on which I shall insist hereafter.

In the third place, we observe that the tongue is thrust out of the mouth by the genio-glossal muscle, whilst the teeth close upon it by the action of the masseters, and it,—or the under lip,—is frequently severely bitten. Or, without the spasmodic protrusion and consequent injury of the tongue, there is grinding of the teeth.

We next observe convulsion,—which is general, or of the whole muscular system, or hemiplegic, or confined to one side; or it occurs in the form of trismus, torticollis, in one limb, &c.

During these attacks, the *expulsors* of the fæces, the urine, or the semen, sometimes act, and there is the unconscious evacuations of these secretions. There is sometimes rigidity of the penis." 145.

Some facts are enumerated as causes of epilepsy—some pathological conditions as effects of it.

The former are set down—deep sleep; broken sleep; loss of rest; passion; vexation; exhaustion; inanition; and especially rising with an empty stomach, have frequently led to a paroxysm of epilepsy, and must, consequently, be carefully avoided in our rules of regimen for the cure of this disease. The act of venery may bring it on. Napoleon, we believe, suffered in this manner. Dr. Hall has known washing the hands in cold water occasion it.

The effects alluded to are the venous congestion of the brain, which results from the very nature of the attack—and the effusion of serum, after many attacks, which results from the venous congestion.

The objects of treatment are two-fold: prevention of the attacks—and prevention of their effects. This is to be done by cautiously avoiding the *causes*, by moderating the *paroxysms*, and by local means of subduing vascular action, and perhaps of depleting the vessels of the brain.

With the following quotation we conclude.

"The strictest rules must be laid down for the diet, for the state of the bowels, for conducting the catamenial periods. These last should be passed in bed, the feet and abdomen should be fomented, the warm water enema, and the opiate enema should be administered.

The immediate accession of the paroxysm may sometimes be prevented by dashing cold water on the face, or by exciting the nostrils by snuff, &c. In this manner the disposition to closure of the larynx, and expiratory efforts, is exchanged for sudden acts of inspiration.

In the paroxysm, the patient must be prevented from injuring himself by falls or blows. In this danger of injury we have another (§610) marked distinction between epilepsy and hysteria.

The stupor or coma induced by the paroxysm may require the administration of blood-letting, general or topical, according to its degree and duration, and probable effects.

Besides the means to which I have alluded, other remedies have been proposed for the cure of epilepsy, in an empirical manner, without due attention to the *kind* of the disease. It is obvious that little attention can be paid to propositions and observations so vague and indefinite. These various remedies must be tried anew, after a strict diagnosis. We shall then arrive at an approximation to the truth in reference to the value of these remedies respectively.

The views which I have given of eccentric epilepsy are amply confirmed by the facts, that there is no constant morbid change observable in this disease, and that many patients, after long years of its attacks, have finally and fully recovered, —facts which ought to encourage us steadily to pursue the mode of treatment.

A system of exercises; regulated sleep; the shower bath; tonic remedies, &c. &c. must be added to the other plans." 147.

Here we must arrest our steps. We have travelled in a desultory manner, sometimes with Mr. Mayo, sometimes with Dr. Hall. Like a courtezan, we have picked the pockets of both, and here are the proceeds of our larcenies.

It was not our intention to offer a systematic account of the pathology of the nervous system. That would be too extensive and too elementary for a Review. Much must be then stated with which even students are familiar, and from which our readers would turn with disgust. We have endeavoured to select in the instances of palsy, apoplexy, concussion and compression of the brain, and epilepsy, some facts not generally known, some opinions deserving of attention, and some theories which require investigation. We have dovetailed as well as we were able, and we would fain hope that there has resulted neither a disagreeable nor an useless article.

Such a plan is more palatable to the public than the author. To cater for the former, the latter must be mutilated, and he cannot see with pleasure his disjecta membra dressed in a strange fashion, and served with foreign sauces. He feels he is but an element in an Olla Podrida, and his vanity is wounded by the insult.

But Dr. Hall and Mr. Mayo may console themselves with the assurance that the affront is not on them, but on the nature of their subjects. The works of both are in a great measure elementary, and professedly addressed to students. We could not therefore do more than select individual parts for criticism or for notice, and much as we should feel inclined to please them both, they know that we are compelled to please our readers.

The pathology of the nerves and of the spinal marrow yet remains for us.* There remains too, a more particular notice of the physiological views of Dr. Hall. One or other of these subjects will certainly engage us at the earliest opportunity. It is pleasant to be in communication with men of genius, as both our authors are. If we differ from them upon any point, we state fairly the grounds of difference, and if we are wrong there are the data for conviction. Dr. Hall remarks, in the volume before us, that he knows that men will dispute. It is right they should, for we may feel assured that in science it is disputation only that can lead to truth. Objections when not disingenuously urged, facts when fairly stated, are the best means for eliciting the right, and for supporting it. Criticism is only powerful when correct—against the fact it is, in the present day, utterly impotent.

---

* We promise ourselves much gratification from an analysis of Sir B. Brodie's forthcoming paper on the Injuries of the Spinal Marrow—a continuation of his paper on the Injuries of the Head.

